# Power Imbalance and the Dark Side of the Captive Agri-food Supplier–Buyer Relationship

**DOI:** 10.1007/s10551-021-04791-7

**Published:** 2021-03-19

**Authors:** Richard Glavee-Geo, Per Engelseth, Arnt Buvik

**Affiliations:** 1 Department of International Business, NTNU-Norwegian University of Science and Technology, P. O. Box 1517, 6025 Aalesund, Norway; 2 School of Business and Economics, University of Tromsø, Narvik Campus, Lodve Langesgate 2, 8514 Narvik, Norway; 3 Faculty of Logistics, Molde University College, P. O. Box 2110, 6402 Molde, Norway

**Keywords:** Buyer control, Buyer opportunism, Buyer power, Power asymmetry, Supplier exploitation, Unethical behaviour

## Abstract

This paper highlights the dark side of power imbalance regarding its consequences in agri-food supplier–buyer relationships. We report on findings from two studies. The first study is based on a sample of 105 key informants, while study 2 is based on a sample of 444 key informants, all from the cocoa agri-food supply market of Ghana. While the first study focuses on the antecedents of power imbalance and its consequences, the second study explores the role of cooperatives/collective action in minimizing supplier exploitation. Data from these studies were analysed using the partial least squares technique (SmartPLS). Analysis of these findings shows switching costs’ impact on power imbalance to be curvilinear, while power imbalance has a curvilinear relationship with opportunism. The negative consequences of power imbalance are further exacerbated by dependency and the lack of joint action. Furthermore, we found the negative impact of power imbalance on financial performance to be stronger for non-cooperative members than for cooperative members, while, counterintuitively, we found the positive impact of economic satisfaction on financial performance to be stronger for non-cooperative members than for cooperative members.

## Introduction

Most agri-food systems in emerging and developing economies are characterized by small suppliers, which are highly dependent on much larger buyers and lead firms. These suppliers have high switching costs and are therefore described as ‘‘captive’’ (Cox et al., 2000). While it is generally known and assumed that power can be misused and can lead to exploitation, the topic of unscrupulous and ethically unprincipled buying behaviour has been ignored in academic research in general (Schleper et al., 2017). Cases of supplier exploitation by firms or their agents have been popular in the media. Power imbalance and dependence have been cited as causing and fostering supplier exploitative and negative behaviours (Abosag et al., 2016; Hingley, 2005b; Schleper et al., 2017). The power concept is critical in understanding buyer–supplier relationships (Gaski, 1984). In supply chain relationships, power is useful for effective coordination, integration, and goal attainment, such that weaker participants in the chain may be predisposed to endure imbalanced relations so long as there are ample rewards (Hingley, 2005a; Maglaras et al., 2015; Nyga et al., 2013). Asymmetries in power affect the distribution of outcomes (Hingley, 2005a).

Grandinetti (2017) identified two different types of dark sides to business relationships, termed “trap” and “secret”. In the first case, the partner who is at a disadvantage is aware of what is going on but nevertheless remains trapped in the relationship because of a power imbalance and a strong dependence. In the second case, one partner exploits an information asymmetry (a secret) to his own advantage and to the other partner’s disadvantage. Our study is focused on the first scenario, where the supplier is aware of what is going on but nevertheless remains trapped in the relationship because of a power imbalance and a strong dependence. In addition, despite the recent widespread use of the term ‘dark side’ in business relationships, little critique of the literature and empirical research has been offered (Abosag et al., 2016, p. 5). In response to the above, and to fill the knowledge gap, the empirical focus is notions of power imbalance in a developing country, looking at the cocoa raw material market, comprising an upstream supply chain marketplace of growers with customers who eventually are tiered within developed marketplaces. Our goal is to highlight the impact of power in business relationships regarding the ‘dark side’ phenomenon and how it erodes the gains of suppliers in agri-food supply chains. The research objectives of this study are several. We seek to examine: (1) how dependency and high switching costs in agri-food supplier–buyer exchanges influence perceptions of power imbalance; (2) the conditions under which power imbalance provide ideal situations for unethical behaviours; (3) the consequences of power imbalance, opportunistic behaviour and decision control; and (4) to provide theoretical contributions, managerial insights and implications.

The Ghanaian cocoa supply market is the empirical context. The paper concerns the relationship between buying agents, who are intermediaries between cocoa growers and the buying firms. Several events have been reported of unscrupulous conduct by various buying agents who undervalue sales from cocoa growers (Dadzie et al., 2018). The financial loss caused by inaccurate weighing of produce can be a source of cognitive distrust (Dadzie et al., 2018). The global cocoa-chocolate market is estimated to reach $140 billion by 2024. Ghana is the second leading exporter of cocoa globally (Dadzie et al., 2018; Oomes et al., 2016). The national supply market has strong interconnections to the global cocoa-chocolate market. The power relations in the Ghanaian context are similar to several cocoa-producing countries, which strengthens the generalizability of our results. The global cocoa-chocolate supply chain is a complex network of cocoa growers, traders, exporters, converters/grinders, chocolate manufacturers, and retail chains. Based on our findings from a quantitative study administered in this industry, we argue that the investment in positive elements by businesses alone is not adequate, as business partners must protect suppliers against detrimental actions and behaviours.

The rest of the article is organized as follows: in the next section, we review the literature and present the research hypotheses. This is followed by the research methods, results, and the discussion. We conclude with the limitations and suggestions for further research.

## Literature Review and Research Hypotheses

### Dark Side of Business Relationships

No business relationships are absolutely light or dark, but instead embody a mix of these two (Abosag et al., 2016). Previous literature (e.g. Håkansson and Snehota, 1998) emphasizes the dark side as deriving from the value of relationships. The literature (e.g. Abosag et al., 2016) has identified various situations that constitute the dark side, such as relationship unrest (Good & Evans, 2001), relationship burdens (Håkansson and Snehota, 1998), relationship stress (Holmlund-Rytkönen & Strandvik, 2005), the adverse sides of business relationships (Strandvik & Holmlund, 2008), relational misconduct (Hawkins et al., 2008), detrimental intentions (Liu et al., 2014), and exploitative business relationships (Schleper et al., 2017).

The concept of a ‘dark side’ to business relationships suggests “‘problems’, ‘challenges’, ‘difficulties’, and ‘drawbacks’ related to structural issues … such as size differences, or the imbalance of power; processes within business relationships, including creativity issues, capability development, changes in market dynamics; and outputs, for example, performance, competitiveness and satisfaction” (Abosag et al., 2016, p. 5). The dark side relates to negative business situations (e.g. Gaski, 1984; John, 1984). Regarding collaboration in service networks, the dark side refers to those aspects of co-creation activities that are hidden and include potential risks in business interactions (Chowdhury et al., 2016). Resource integration activities lead to value co-creation; however, particular relevance is the co-destruction of value which aligns with the dark side of business relationships. This assertion comes from the recognition that engaging in value co-creation may not always lead to symmetrical value outcome or desirable outcome (Dong et al., 2008; Chowdhury et al., 2016). Value can be co-destroyed when resources are accidentally or intentionally misused (Plé & Cáceres, 2010). Moving beyond power and dependence in dyadic relationships to a focus on triads and the wider network in supply chains, we argue that cooperative and joint actions have the potential of limiting the undesirable effects of the dark side. This is because even close dyadic relationships that seem to be the most stable are vulnerable to the dark side phenomenon (Anderson & Jap, 2005). The light side implies that positive benefits and desirable outcomes are derived from the relationship; it denotes the situation in which good relationship quality yields achievable relationship functions and performance (Fang et al., 2011).

### Theories of Power

Power is the ability of one channel member (A) to get another channel member (B) to do something that it otherwise would not have done (Coughlan et al., 2006; Dahl, 1957; Gaski, 1984). It refers to the capability of a partner in an exchange to induce another partner to change its behaviour in favour of the objectives of the partner exerting the influence (Wilemon, 1972). The association between power and dependence is better understood from Emerson's (1962) theory. We use ‘dependence’ and ‘dependency’ interchangeably with the same meaning and intended purpose. Dependency exists between two channel members when the benefits derived from their relationship are not available outside it. Thus, A’s power over B increases with B’s dependence on A. In imbalanced power relationships, the weaker firm is highly dependent on a more powerful firm or its agent to continue to achieve its goals. Whenever one partner (i.e. the principal) depends on another (i.e. the agent) to undertake some action on the principal’s behalf, this leads to a principal–agent relationship (Eisenhardt, 1989; Fontrodona & Sison, 2006). Power and dependence in exchange relationships lead to vulnerability, and the possibility of one partner or its agent being opportunistic and unethical. Dependence is a function of the greater utility (i.e. value, benefits, satisfaction) that B gets from A and the fewer alternative sources of that utility that B can find. It has to do with how easily B can replace A. The dependence condition creates a relational trap in which the weak partner has no choice but to endure the opportunism of the other (Grandinetti, 2017).

To varying extents, firms always depend on their trading partner. Early studies on dependence focused on the effects for the buyer of its dependence on the supplier, without considering the supplier’s dependency (e.g. El-Ansary & Stern, 1972). A few studies have incorporated dependence from the perspective of both the buyer and the supplier (Buchanan, 1992; Geyskens et al., 1996; Kumar et al., 1995). Dependence is defined, in psychology and social psychology, as a state in which assistance from others in terms of finance, emotion, protection, security or daily care, is expected or actively sought (Zhou et al., 2007). In the context of buyer–seller relationships and channel studies, dependence refers to the extent to which a trade partner provides important and critical resources for which there are few alternative sources of supply (Buchanan, 1992), or “the degree to which the target firm needs to maintain its relationship with the source in order to achieve its desired goals” (Kale, 1986, p. 390).

### Research Hypotheses

#### Dependence, Power Imbalance and Financial Performance

In asymmetric relationships, the more independent partner dominates the exchange. Balanced relationships refer to domination by neither party (Buchanan, 1992). Kumar et al. (1995) use the term ‘interdependence asymmetry’, defined as the difference between the two partners’ levels of dependence. Symmetrical interdependence exists when parties are equally dependent on one another. In asymmetric interdependence, the independent partner experiences high power and might be attempted to exploit it (Geyskens et al., 1996). Therefore, power imbalances within a buyer–supplier relationship can lead to unproductive partnerships (McDonald, 1999) and hence low economic benefits. In most buyer–seller relationships, disparities in turnover or values of sales and the size of the supplier compared to the buying company place the supplier in a less powerful exchange position. The buying companies are more powerful and have the leading role in respect of relationship administration and allocation of rewards (Griffith et al., 2006). Furthermore, the annual value of total sales from a supplier to a buyer is an indication of the economic rewards that the supplier receives for the sacrifice of forgoing alternative exchange relationships. Based on the theory review and reasoning proffered here, we posit that:

##### H1a

The effect of supplier dependency on power imbalance is moderated by transaction/sales volume.

##### H1b

The effect of power imbalance on financial performance is moderated by supplier dependency.

#### Effect of Switching Costs on Power Imbalance

An imbalance of power within the buyer–supplier relationship may lead not only to dissension by the offended partner but to a low performance by the aggrieved partner. In such situations, the social capital built up within the relationship may deteriorate. Switching costs, defined as the need to maintain a relationship due to latent costs that would be incurred if that relationship were to end (Scheer et al., 2015), is a key variable impacting on dependency and hence performance. Partners’ investment in terms of time, effort, and money, along with perceived costs of switching, can contribute to dependency (Emerson, 1962; Scheer et al., 2015) and hence high perceptions of power imbalance. Supplier-perceived relationship value had been found to be negatively related to intention to switch, while support for the negative association between switching costs and switching intention has been supported (Geiger et al., 2012). Dabholkar et al. (1994) paper on the dynamics of long-term business-to-business exchange relationships suggests that exchange relationships are formed by achieving mutually beneficial outcomes from a series of exchange transactions. The authors propose a curvilinear relationship between relative power and gains (relationship outcomes) at high switching costs levels. Many micro and small businesses dealing with the more powerful large firms lack the resources to seek alternative business relationships. The more difficult it is for, the weaker partner to switch, the more vulnerable partner perceives the power differential. The more powerful and dominant firm begins the exchange relationship from a power advantage; however, as the exchange relationship develops from transactional to closer and long-term mutual relationship, power imbalance impact diminishes. However, as it becomes more difficult for the weaker partner to switch due to high dependency on the more powerful partner, the two partner's power differential becomes more obvious temporally. Therefore, we expect a U-shaped relationship between switching costs and power imbalance. Based on the above argument, we hypothesize that in a captive supplier–buyer relationship:

##### H2a

Switching costs are associated with increased power imbalance.

##### H2b

The association between switching costs and power imbalance could be curvilinear.

#### Effect of Power Imbalance on Opportunism

The risk of opportunistic behaviour creates uncertainty, thereby requiring some level of cooperative effort to ensure a successful relationship (Rokkan & Buvik, 2003). Opportunism refers to a self-seeking phenomenon characterized by exploitation (John, 1984; Williamson, 1985). Numerous examples of opportunistic behaviour have been documented. A few examples include salespeople exaggerating expenditure reports (Philips, 1982); resellers violating explicit resale agreements (Dutta et al., 1994); physicians prescribing excessive quantities of expensive drugs to patients (The Economist, 1996); and inaccurate weighing of produce by buying agents (Dadzie et al., 2018). It is logical to assume that the power differential between trading partners could provide a conducive environment for opportunistic and unethical behaviour (Ireland and Webb, 2007). A dependence power advantage is one of the most cited antecedents of opportunism in the inter-firm relationship literature (Grandinetti, 2017; Hawkins et al., 2008; Tangpong et al., 2015; Wang & Yang, 2013). Previous studies had shown that opportunism’s association with other variables could be curvilinear, for example, opportunism and performance (Lado et al., 2008), opportunism and goal exceedance (El Meladi et al., 2018), opportunism and punishment severity (Xiao et al., 2019), and opportunism and guanxi (Shen et al., 2019). Drawing on the long-term perspective of Dabholkar et al. (1994) and the relationship development cycle (Dwyer et al., 1987), opportunism is expected to be low at the relationship initiation stage but, as the relationship develops and power relations become obvious, the partner or its agent with the higher power could easily use the power advantage to exploit the weaker partner. Consequently, we expect an inverted U-shaped relationship between power imbalance and opportunism. In view of the above deliberations, we hypothesize that:

##### H3a

Power imbalance is associated with increased opportunism.

##### H3b

The association between power imbalance and opportunism could be curvilinear.

#### Effects of Power Imbalance and Opportunism on Financial Performance

Power plays an important role in the supply chain, judicious use of power may serve to benefit the power holder (Benton & Maloni, 2005). Lavan (2007) suggests that cocoa farmers in Ghana do not benefit much economically from their farm businesses because of their inability to organize themselves. The suppliers lack a strong voice with the requisite bargaining power to negotiate with the state regulator and the buying firms they deal with. Hence, it is expected that cooperative marketing membership could increase the bargaining power of suppliers to counteract the disadvantages associated with a power imbalance. We define a cooperative as the use of collective action through participation in a voluntary venture by a group of individuals or independent enterprises with the aim of achieving benefits through the coordination of activities such as logistics, purchasing, and marketing. In supplier–buyer relationships, gains accruing to exchange partners reflect how profitable the exchange process has been regarding performance. Exploitation of the relative power of the buying company or its personnel/agent is therefore expected to lead to dissatisfaction and reduce financial gains for the weaker partner. Small-scale farm businesses can collaborate to diminish the influence or unscrupulousness of intermediaries and businesses. Meier zu Selhausen (2016) suggests that active involvement in agricultural marketing and collaborative efforts help less dominant suppliers to overcome exploitation. In line with the above reasoning, we hypothesize that in a captive supplier–buyer relationship:

##### H4a

Power imbalance is associated with reduced financial performance.

##### H4b

The effect of power imbalance on reducing financial performance is stronger for non-cooperative members than for cooperative members.

##### H4c

Buyer/agent opportunism is associated with reduced supplier financial performance.

#### Effects of Buyer Control of Price and Quality on Financial Performance

Decision control in terms of buyer control has been conceptualized as the extent to which the buyer has authority and control over supplier decision-making (Buvik & Andersen, 2015). It refers to centralization of the decision-making authority. In this study, buyer control refers to the ability of one partner to influence and control the other in relation to channel activities. Control as a governance mechanism is used to regulate trading and overcome performance measurement complexities linked to mutual dependence (Williamson, 1985). Supplier dependency on the buyer creates power imbalance situations where the shift in power favours the buyer. The power differential augments the buyer’s ability to control the decisions of the supplier (Anderson & Weitz, 1989; Emerson, 1962; Joshi, 1998). Price control by the power source could lead to low gains for weaker channel members (Belaya & Hanf, 2009; Hingley, 2005a; Maglaras et al., 2015). However, control of quality through standardization could lead to beneficial outcomes. The Japanese are known for their emphasis on quality, such that suppliers of large manufacturers such as Toyota must meet stringent quality standards to improve performance. In demanding quality improvements from vendors, in many cases companies will use only suppliers that have passed the expensive and time-consuming ISO 9000 certification (Krause et al., 2007). Based on the above reasoning, we hypothesize that in a captive supplier–buyer relationship:

##### H5a

Buyer control of price is associated with reduced financial performance.

##### H5b

Quality standardization is associated with increased financial performance.

##### H5c

The effect of power imbalance on financial performance is moderated by quality standardization.

#### Effect of Economic Satisfaction on Financial Performance

Supplier satisfaction is defined as a supplier’s cognitive and affective state of feeling sufficiently rewarded economically and psychosocially for the sacrifices undergone in facilitating the exchange relationship, no matter what power imbalance may exist between the supplier and the buyer (Benton & Maloni, 2005). In this study, we consider satisfaction as a two-dimensional construct consisting of economic and non-economic or social satisfaction (Ferro et al., 2016; Geyskens & Steenkamp, 2000; Rodriguez et al., 2006). Economic satisfaction refers to a channel member’s evaluation of the economic outcomes that result from the relationship, while non-economic or social satisfaction refers to the psychosocial, non-economic aspects of the relationship, in that interaction with the exchange partner is fulfilling, gratifying, and characterized by tranquillity (Geyskens et al., 1999). Social exchange theory posits that exchange may involve both economic and social outcomes. The need for “consistent delivery of economic and psychosocial benefits in each transaction” within the exchange process is critical for the sustenance of relational exchanges (Dwyer et al., 1987, p. 25). A supplier that is satisfied economically evaluates the relationship as being successful in terms of goal attainment, relationship effectiveness and productivity (Geyskens et al., 1999), including financial results. An exchange partner’s perception of being sufficiently rewarded economically is key in imbalanced business relationships. The use of collective action through participation in marketing cooperatives is expected to offset asymmetrical power relations and to improve the welfare of its members. We hypothesize that in a captive supplier–buyer relationship:

##### H6a

Economic satisfaction is associated with increased financial performance.

##### H6b

The effect of economic satisfaction on financial performance is stronger for cooperative members than for non-cooperative members.

## Research Methods

### Research Setting and Data Collection

The context is the cocoa industry of Ghana. Certain cocoa-producing countries have a fully liberated local market with a free-market system characterized by many private exporters. While in others (e.g. Ghana) private, former state marketing monopolies retain substantial control and play ‘coordinative’ role in chain governance (Glavee-Geo, 2019). In Ghana, the industry is partially liberated, characterized by the participation of private firms and many cocoa growers as the main suppliers. The industry regulator is the Ghana Cocoa Board (COCOBOD).

We collected primary data from the cocoa growers, who were very knowledgeable about the issues at stake. The respondents were either farm owners or farm managers serving as key informants. We based the sampling on farms located in the southern part of Ghana according to the knowledge of the industry regulator. Approval was sought from each informant before each interview. Subsequently, primary data were collected through face-to-face interviews. In most developing and some emerging countries, data collection through mail or email leads to low response rates—hence the need for some other innovative means of data collection through household interviews. The lead author conducted the interviews for study 1 over a period of two weeks and for study 2 a year later for a period of five weeks in the cocoa-growing regions of Central, Eastern, and Ashanti regions of Ghana. The respondents were mostly farm owners who were interviewed in their houses (sometimes with a farm manager providing corroborative information). Study 1 consisted of 105 respondents while study 2 consisted of 444 respondents.

### Operational Measures

All constructs are based on reflective multi-item scales adapted from previously validated scales (see Tables 1 and 2). The indicators are measured on a seven-point Likert scale, with 1 representing the lowest level (strongly disagree) and 7 the highest level (strongly agree). Subjective measures of supplier financial performance are newly formulated, while the profitability measure is adapted from Haugland et al. (2007). The difficulty in accessing financial data makes the use of subjective measures of performance a better proxy to evaluate the performance of a supplying firm. The three-item scale of supplier financial performance is formulated with the anchors 1, representing worse performance, and 7, representing better performance. Single-item indicators of the relative size of the buyer, relationship duration, transaction/sales volume, supplier dependency, switching costs and buyer control of price were formulated based on the extant literature (e.g. Buvik & Andersen, 2015; Caniels & Gelderman, 2007). A single item—‘this buying company ensures that the minimum producer price does not vary’—measured buyer control of price. Supplier dependency was measured with the question ‘how large do you perceive your dependency on this particular buying company compared to other buying companies within this district?’ Switching costs were measured with ‘how much will it cost you if you want to replace this buying company with another one in a new location?’ The quality standardization item was formulated based on buyer control measures by Buvik and Andersen (2015). Power imbalance items were adapted from Joshi and Stump (1999). In study 1, power imbalance items were operationalized based on two main questions: ‘how large were your sales to this buying company?’ and ‘how large do you perceive the cash bonuses paid by this buying company to your farm business?’ However, in study 2, we sought to improve measurement of the power imbalance through reformulation of the two additional questions from study 1. Reliability results of studies 1 and 2 show the power imbalance scales had high internal consistency and extracted more than 50% of the variance in each instance (see Tables 1 and 2). Membership of cooperatives in study 2 was measured with the question ‘are you a member of any cooperative farmers association?’ with responses coded as a dummy, 1 for ‘yes’ and 0 for ‘no’. Opportunism items were adapted from John (1984) and Skarmeas et al. (2002). Five items were used in study 1, while seven items were used in study 2 with the same intended purpose of achieving good internal consistency. Economic satisfaction items were adapted from Geyskens and Steenkamp (2000) and Skinner et al. (1992), social satisfaction items from Benton and Maloni (2005) and Crosby et al. (1990).

**Table 1:** Construct, reliability, average variance extracted, descriptive statistics, loadings, *t*-values (*n* = 105)

Construct	Indicators	M	SD	Loadings	*t*-value #
Power imbalance CR = 0.90 *α* = 0.78 AVE = 0.82	With respect to sales volume during the last twelve months…				
	How large were your sales to this buying company?	5.67	1.58	0.938	23.840***
	How large do you perceive cash bonuses paid by this buying company to your farm business?	5.58	1.26	0.870	19.163***
Buyer/agent opportunism CR = 0.84 *α* = 0.77 AVE = 0.52	The buying company often undervalues my cocoa beans	4.24	2.13	0.507	2.962**
	The buying company often underrates the quality of my cocoa beans	4.29	2.11	0.842	5.939***
	The buying company often neglects to correct sales’ errors in my transactions	4.47	2.13	0.801	5.645***
	The buying company often weighs my cocoa beans as less than their actual weight.	3.78	1.92	0.777	5.786***
	The buying company often pays less cash bonus than I deserve	3.43	2.26	0.627	4.180***
Supplier financial performance CR = 0.90 *α* = 0.83 AVE = 0.75	Compared to other farm businesses, my farm business has performed relatively well during the last six months in the following respects				
	Profitability	5.86	1.32	0.881	30.787***
	Return on investment	5.88	1.26	0.909	37.774***
	Debt repayment	5.77	1.14	0.798	17.773***
Quality standardization CR = 0.88 *α* = 0.83 AVE = 0.65	This buyer makes sure the quality of the cocoa I sell is okay before taking possession	5.99	1.05	0.919	8.569***
	This buyer takes control of the product for quality inspection	5.96	1.20	0.733	6.629***
	This buyer ensures that the quality test is passed	5.93	1.03	0.773	4.865***
	This buyer always rejects poor quality cocoa sold to their company	5.89	1.31	0.798	4.458***
Economic satisfaction CR = 0.86 *α* = 0.76 AVE = 0.67	My relationship with this buying company is very attractive with respect to prompt payment of cash bonuses	5.49	1.65	0.824	14.788***
	I am very pleased with my decision to sell to this buyer due to the financial benefits the company provides my farm business	5.88	1.22	0.837	18.205***
	I am very satisfied with the price at which I sell my cocoa to this buying company	5.50	1.54	0.802	12.971***
Social satisfaction CR = 0.91 *α* = 0.86 AVE = 0.77	I am satisfied with dealing with this buying company	5.73	1.57	0.909	11.636***
	I would always continue selling to this buying company because of the good personal relationship I have with the staff	5.87	1.59	0.810	7.131***
	I am always pleased to deal with this buying company	6.10	1.28	0.919	13.212***

*CR* composite reliability, *α* Cronbach’s alpha, *AVE* average variance extracted, *M* mean, *SD* standard deviation

^#^Based on 5000 bootstrapping samples: ***significant at *p* < 0.001, **significant at *p* < 0.01 (two-tailed test)

**Table 2:** Construct, reliability, average variance extracted, descriptive statistics, loadings, *t*-values (*n* = 444)

Construct	Indicators	M	SD	Loadings	*t*-value #
Power imbalance CR = 0.90 *α* = 0.86 AVE = 0.70	The buying company enjoys more power in our relationship owing to my small sales volume	5.38	1.43	0.816	28.385***
	The buying company enjoys more power in our relationship because the volume percentage supplied is small	5.50	1.36	0.903	68.690***
	The buying company enjoys more power in our relationship because my cash bonus percentage is small	5.47	1.44	0.816	31.346***
	Overall, this buying firm has more power than my farm business	5.60	1.44	0.806	27.437***
Buyer/agent opportunism CR = 0.87 *α* = 0.86 AVE = 0.81	The buying company often pays less cash for the supplied cocoa beans	4.38	1.92	0.878	59.082***
	The buying company often pays less cash bonus than I deserve	4.62	1.89	0.925	76.581***
	The buying company often undervalues my cocoa beans	4.68	1.94	0.903	72.195***
	The buying company often underrates the quality of my cocoa beans	4.31	1.93	0.904	71.480***
	The buying company often neglects to correct sales’ errors in my transactions	4.41	1.86	0.912	82.731***
	The buying company often weighs my cocoa beans as less than their actual weight	4.58	1.86	0.882	58.423***
	Overall, the buying company often pays less cash bonus than I deserve	5.60	1.92	0.912	86.376***
Supplier financial performance CR = 0.93 *α* = 0.89 AVE = 0.82	Compared to other farm businesses ,my farm business has performed relatively well during the last six months in the following respects				
	Profitability	4.67	1.48	0.917	100.236***
	Return on investment	4.78	1.45	0.925	105.192***
	Debt repayment	4.83	1.47	0.870	43.712***
Economic satisfaction CR = 0.93 *α* = 0.90 AVE = 0.72	My relationship with this buying company has been very beneficial to my farm enterprise	5.39	1.32	0.783	27.365***
	My relationship with this buying company is very attractive concerning prompt payment of cash bonuses	5.34	1.44	0.881	60.071***
	I am very pleased with my decision to sell to this buyer due to the financial benefits in the form of soft loans	5.27	1.45	0.896	81.205***
	I would recommend that other farmers sell their products to this buying company to benefit financially	5.13	1.46	0.886	87.448***
	I am always very satisfied with the amount of cash bonus paid to me by this buying company	4.91	1.51	0.807	41.630***
Social satisfaction CR = 0.96 *α* = 0.94 AVE = 0.81	I have a favourable relationship with this buying company’s personnel	4.76	1.49	0.849	45.231***
	I am satisfied with dealing with this buying company	4.75	1.58	0.904	82.119***
	I would always continue selling to this buying company because of the good personal relationship I have with the staff	4.67	1.62	0.926	127.786***
	This buying company is good to do business with	4.65	1.65	0.918	109.815***
	I am always pleased to deal with this buying company	4.71	1.61	0.910	100.417***

*CR* composite reliability, *α* Cronbach’s alpha, *AVE* average variance extracted, *M* mean, *SD* standard deviation

^#^Based on 10,000 bootstrapping samples: ***significant at *p* < 0.001 (two-tailed test)

### Estimation

We conducted the estimation using the partial least squares (PLS) structural equation modelling technique SmartPLS 3.0 (Ringle et al., 2015). PLS’ strength lies in its ability to deal with complex models with a high number of constructs, indicators and relationships (Hair et al., 2017). It is less strict with assumptions about the distribution of the data and equally ideal for small sample size (Chin & Newsted, 1999; Hair et al., 2017). The use of categorical variables with unknown non-normal frequency distribution, which are usually negatively skewed, makes PLS preferable. The preceding factors make it an ideal analytical technique for the current study and hence a preferable alternative to the use of maximum likelihood methods.

## Results

### Descriptive Statistics, Measurement Reliability and Validity

The descriptive statistics of the variables (mean and standard deviation), factor loadings, reliability, and average variance extracted (AVE) of the constructs for study 1 and 2 are presented in Tables 1 and 2.

The standardized loadings for the indicators and bootstrap t-values for all the items used in both study 1 and study 2 (see Tables 1 and 2) were all significant at 0.001 in the two-tailed test, except the item ‘The buying company often undervalues my cocoa beans’, which is significant at *p* < 0.01 (Table 1). All Cronbach’s alphas exceed the 0.7 threshold (Nunnally, 1978), while composite reliabilities (Fornell & Larcker, 1981) are higher than 0.8, showing high internal consistency. The average variance extracted (AVE) (Fornell & Larcker, 1981) exceeds 0.50, the lowest being 0.52 for the construct buyer/agent opportunism in study 1 and the highest 0.82 for the constructs supplier financial performance (study 2) and power imbalance (study 1) (see Tables 1 and 2). A higher AVE indicates that the variance captured by each latent variable is significantly larger than the variance due to measurement error, demonstrating unidimensionality and high convergent validity of the constructs.

We assessed discriminant validity (Chin, 1998; Fornell & Larcker, 1981) by comparing the square root of the AVE for each construct with the correlations of all other constructs in the model (see Tables 3 and 4). A correlation between constructs exceeding the square roots of their AVE indicates that they may not be sufficiently discriminable (Coelho & Henseler, 2012; Hair et al., 2017). A comparison of the square root of the AVE (bold face diagonal values) and the correlations among the constructs shows that the square roots of AVE are always higher than the absolute correlations between the constructs. We also checked the heterotrait–monotrait ratio of correlations and found all the values were under 0.85, demonstrating high discriminant validity (Henseler et al., 2015). We conclude that the measurement models for study 1 and study 2 show evidence of acceptable validity.

**Table 3:** Discriminant validity coefficients (*n* = 105)

	1	2	3	4	5	6	7	8	9	10	11	12
Buyer control of price (1)	1.00											
Quality standardization (2)	0.00	**0.81**										
Economic satisfaction (3)	0.16	0.28	**0.82**									
Power imbalance (4)	0.19	− 0.06	− 0.19	**0.91**								
Relationship duration (5)	− 0.16	0.14	0.06	− 0.02	1.00							
Social satisfaction (6)	0.11	0.30	0.65	0.02	0.11	**0.88**						
Supplier financial performance (7)	− 0.16	0.39	0.46	− 0.31	0.04	0.21	**0.86**					
Switching costs (8)	0.04	− 0.40	− 0.22	0.21	− 0.08	− 0.18	− 0.33	1.00				
Relative size of buyer (9)	0.08	− 0.15	− 0.15	0.16	− 0.10	− 0.21	− 0.22	0.30	1.00			
Sales volume (10)	− 0.07	0.51	0.06	− 0.04	0.23	− 0.02	0.23	− 0.12	0.05	1.00		
Supplier dependency (11)	− 0.13	− 0.15	− 0.02	0.19	0.10	− 0.06	− 0.21	0.38	0.25	0.01	1.00	
Buyer/agent opportunism (12)	− 0.04	− 0.16	0.32	0.16	0.01	0.27	0.22	0.09	− 0.11	− 0.21	0.04	**0.72**

Bold numbers on the diagonals shows the square root of the AVE; numbers below the diagonal represent construct correlations. Single measure constructs have an average variance extracted of one, with the assumption that they fully measure the latent variable

**Table 4:** Discriminant validity coefficients (*n* = 444)

	1	2	3	4	5	6	7
Economic satisfaction (1)	**0.85**						
Power imbalance (2)	0.02	**0.83**					
Relationship duration (3)	0.17	− 0.02	1.00				
Social satisfaction (4)	0.61	− 0.11	0.09	**0.90**			
Supplier financial performance (5)	0.36	− 0.31	0.30	0.33	**0.90**		
Sales volume (6)	0.20	− 0.04	0.30	0.27	0.64	1.00	
Buyer/agent opportunism (7)	0.27	0.18	0.02	0.29	0.04	0.05	**0.90**

Bold numbers on the diagonals shows the square root of the AVE; numbers below the diagonal represent construct correlations. Single measure constructs have an average variance extracted of one, with the assumption that they fully measure the latent variable

### Common Method Variance

Common method bias (CMV) is variance attributable to the measurement method rather than to the constructs, this is because the data for all the model variables came from the same respondents at the same time. CMV might influence some of the hypothesized relations in the structural model (Podsakoff et al., 2003; Podsakoff & Organ, 1986). To avoid bias from CMV, we applied an a priori method (Hulland et al., 2018). This was done by careful design of the questionnaire and formulation of the question instruments. During the data collection, we conducted the interviews for both studies by asking the questions in a random order: this ensured that the dependent and independent variables in the survey were separated. Before the administration of both surveys, we pre-tested the questionnaire. The pre-test helped us avoid ambiguous question items that could be difficult to understand or interpret. Taking these steps prior to administering the survey helped to limit the potential for CMV.

### Structural Model Estimation Results

The structural model results of study 1 (*n* = 105) and study 2 (*n* = 444) were estimated using SmartPLS 3 (Ringle et al., 2015). To evaluate the structural models of both studies, we first assessed the structural models for collinearity (Hair et al., 2017) by examining the variance inflation factor (VIF) values of all the predictor constructs. We found all the VIF values to be below the threshold of 3.3 (Diamantopoulos & Siguaw, 2006). We concluded that collinearity was not at critical levels (Table 5). Thereafter, we examined the significance and relevance of the path coefficients based on the results of the bootstrapping procedure with 10,000 subsamples (Franke & Sarstedt, 2019).

**Table 5:** Structural model results, effect sizes (*f*^2^) and collinearity (*VIF*) (*n* = 105)

Criterion	*R*^2^	Predictors	Path coefficient	*t*-values#	_*f*2_	*VIF*
Power imbalance	0.17	Switching costs	0.10	0.95	0.01	1.36
		Supplier dependency	− 0.04	0.39	0.00	1.50
		Relationship duration	0.09	1.13	0.01	1.22
		Sales volume	− 0.07	0.64	0.01	1.16
		Relative size of buyer	0.16	1.63^a^	0.02	1.26
		Supplier dependency × sales volume	0.45	2.93**	0.10	1.40
		Switching costs × switching costs	0.17	2.52*	0.06	1.50
Buyer/agent opportunism	0.17	Power imbalance	− 0.17	1.01	0.02	1.83
		Power imbalance × power imbalance	− 0.28	3.71***	0.17	1.83
Supplier financial performance	0.54	Power imbalance	− 0.24	2.65**	0.09	1.41
		Buyer/agent opportunism	0.07	0.75	0.01	1.62
		Quality standardization	0.28	2.76**	0.13	1.40
		Buyer control of price	− 0.18	2.69**	0.06	1.21
		Economic satisfaction	0.43	3.49***	0.17	2.32
		Social satisfaction	− 0.05	0.46	0.00	2.12
		Supplier dependency	− 0.12	1.57^a^	0.02	1.40
		Switching costs	0.08	0.92	0.01	1.69
		Power imbalance × supplier dependency	− 0.23	2.49*	0.06	1.38
		Power imbalance × quality standardization	0.27	3.17**	0.14	1.33
Quality standardization^c^		Power imbalance	− 0.06	0.49	0.00	1.00
Buyer control of price^c^		Power imbalance	0.19	2.24*	0.04	1.00
Economic satisfaction^c^		Power imbalance	− 0.18	1.92^b^	0.03	1.00
Social satisfaction^c^		Power imbalance	0.03	0.22	0.00	1.00

^a^Significant at *p* < 0.10 (one-tailed test)

^b^Significant at *p* < 0.05 (one-tailed test)

^c^*R*^2^ values are negligible

^#^Based on 5000 bootstrapping samples: ***significant at *p* < 0.001 level (two-tailed test); **significant at *p* < 0.01 (two-tailed test); *significant at *p* < 0.05 (two-tailed test)

#### Dependence, Power Imbalance, and Financial Performance

Our analysis shows support for the interaction effect between supplier dependency and sales volume (H1a: *β* = 0.45, *t* = 2.93, *p* < 0.01) and hence provides empirical support for the moderating effect of transaction volume between supplier dependency and power imbalance. In addition, we found support for H1b (*β* = − 0.23, *t* = 2.49, *p* < 0.05) and conclude that the effect of power imbalance on financial performance is moderated by supplier dependency (see Table 5).

**Fig. 1:**
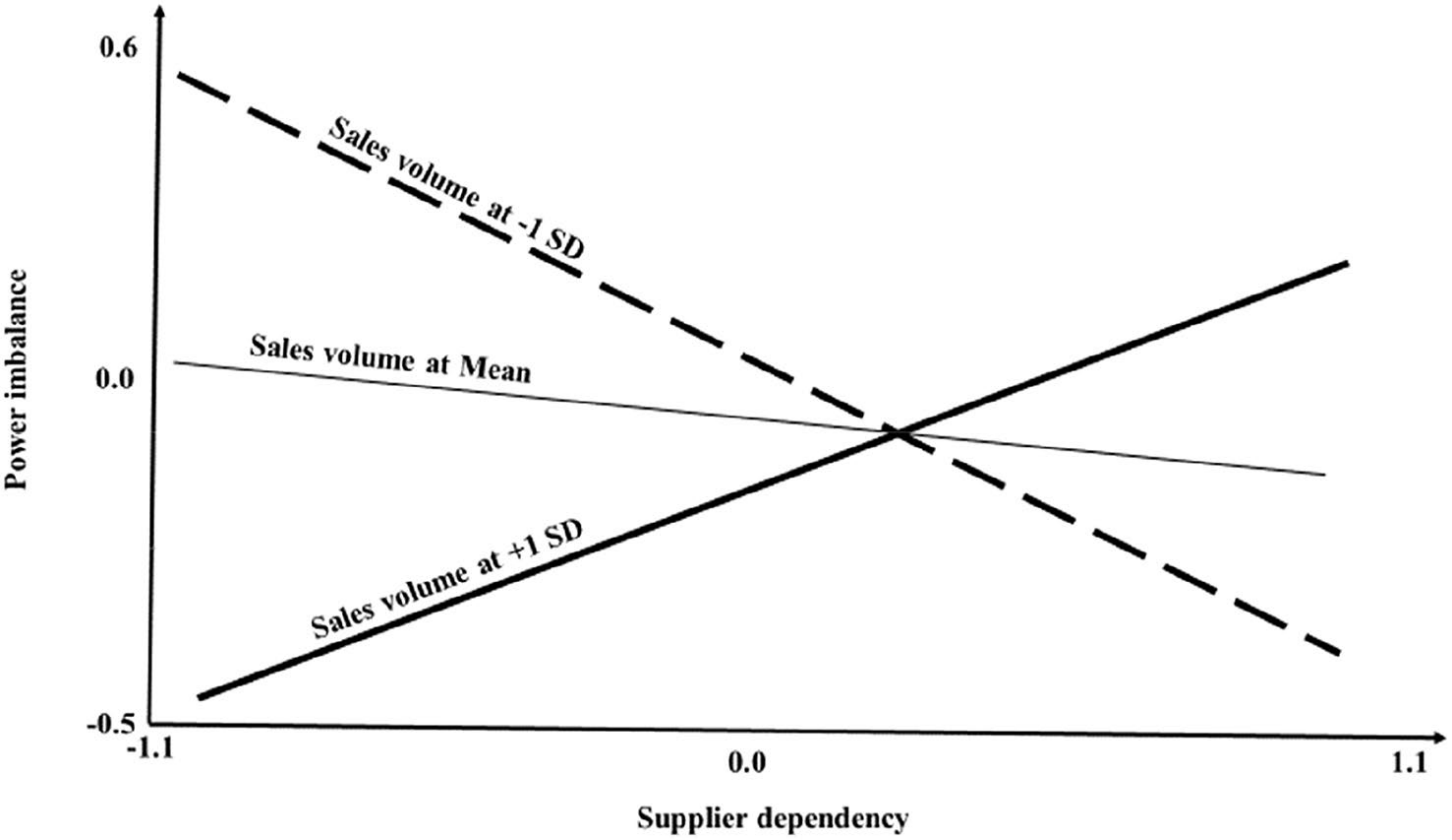
Simple slope analysis of the effect of supplier dependency on power imbalance at various levels of sales volume

**Fig. 2:**
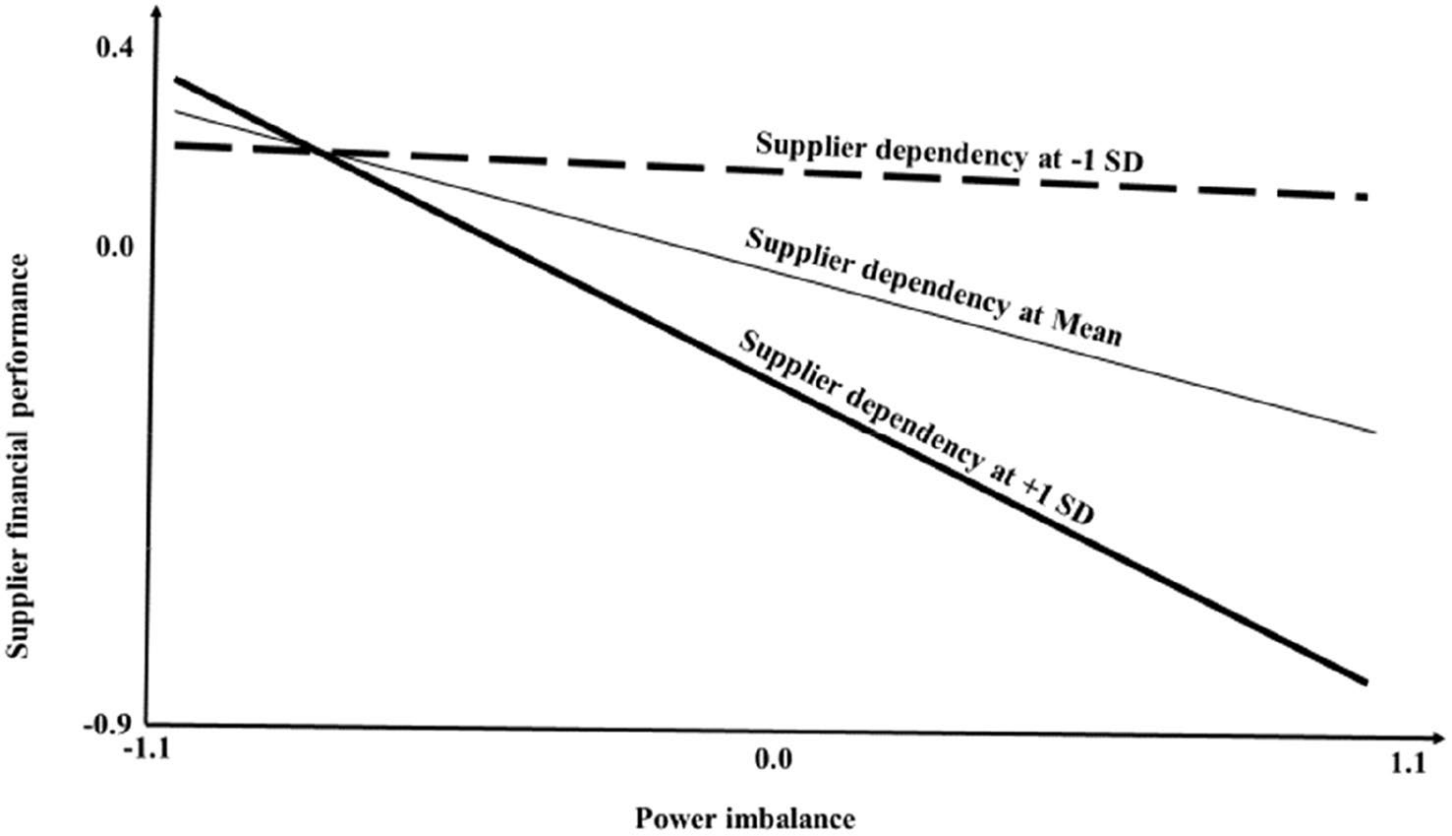
Simple slope analysis of the effect of power imbalance on supplier financial performance at various levels of supplier dependency

Graphical representation of the support for H1a and H1b is shown in Figs. 1 and 2. The simple slope analysis of the effect of supplier dependency on power imbalance at various levels of transaction volume shows that for suppliers of large volumes of sales, dependency increases perceptions of power imbalance, while for suppliers of small transaction volumes, the opposite applies. Figure 2 illustrate the simple slope analysis of the effect of power imbalance on supplier financial performance at various levels of supplier dependency. Figure 2 shows that at high levels of dependency, power imbalance has a negative effect on financial performance.

#### Switching costs, power imbalance, and opportunism

**Fig. 3:**
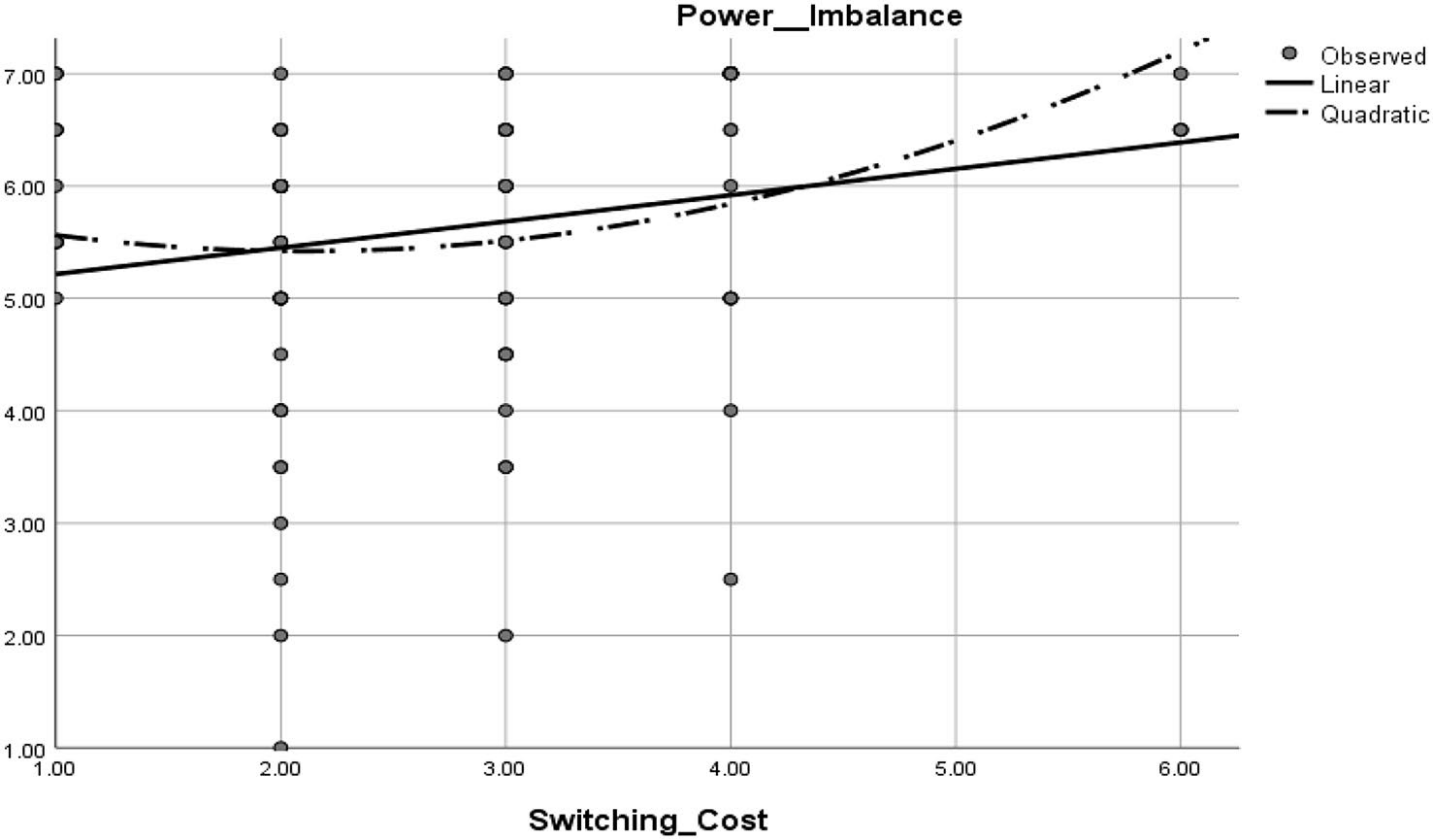
Linear and quadratic slope analysis of the effect of switching costs on power imbalance

We did not find support for H2a, which states that switching costs are associated with increased power imbalance (H2a: *β* = 0.10, *t* = 0.95, p > 0.05); however, we found support for H2b, which shows that the association between switching costs and power imbalance could be curvilinear. Figure 3 illustrates the quadratic slope analysis of the effect of switching costs on power imbalance.

**Fig. 4:**
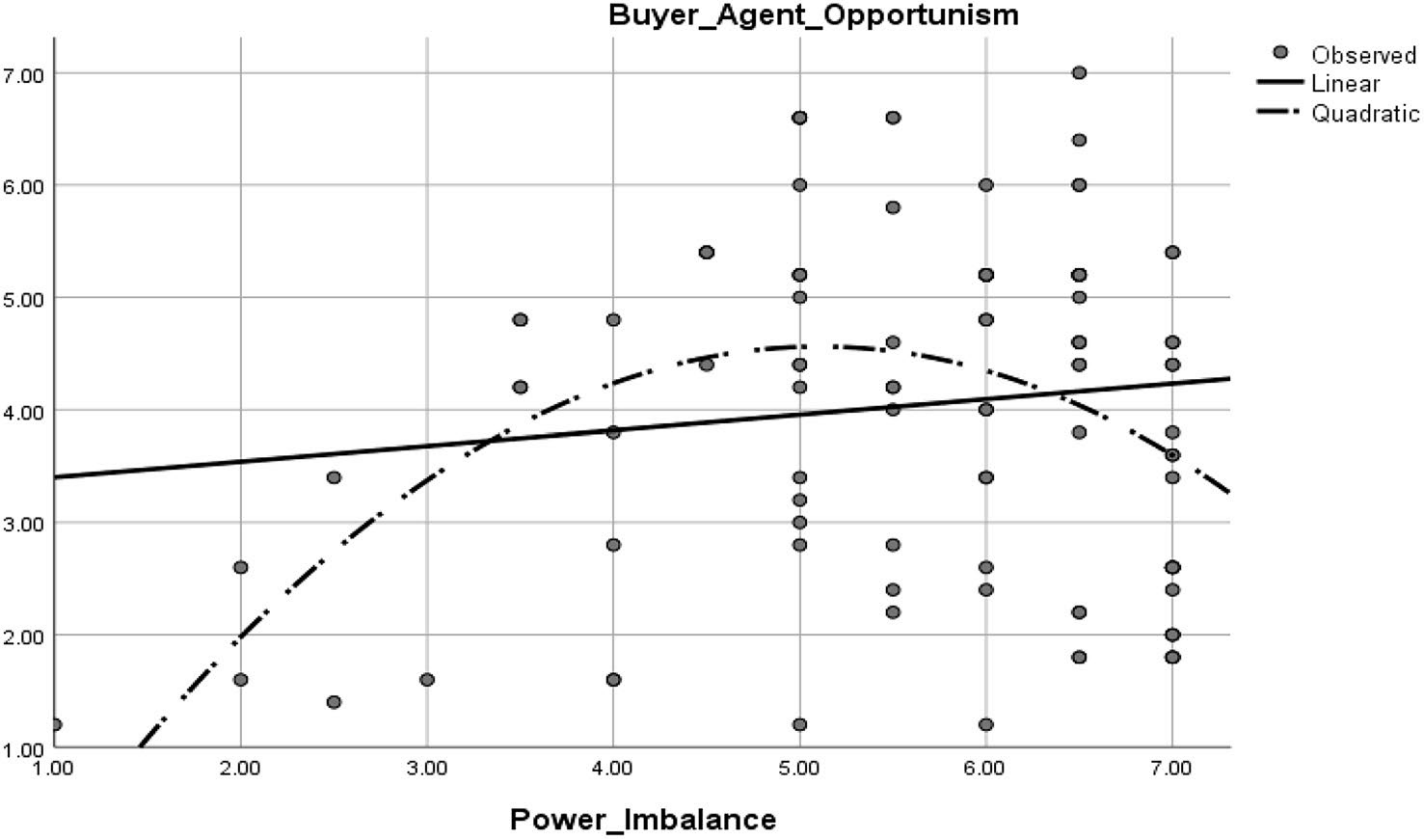
Linear and quadratic slope analysis of the effect of power imbalance on buyer/agent opportunism

Hypothesis 3a states that power imbalance is associated with increased opportunism: in other words, power imbalance is positively associated with opportunism. H3a was supported by study 2 (see Table 6). Furthermore, our results from study 1 (see Table 5) provide support for H3b, which posits that the association between power imbalance and opportunism could be curvilinear (*β* = − 0.28, *t* = 3.71, *p* < 0.001). Figure 4 shows the inverted U-shaped curve which illustrates that the effect of power imbalance on opportunism increases to a point and then decreases.

**Table 6:** Structural model results and t-statistic for multigroup analysis

Criterion	Predictors	Combined (*n* = 444)		Non-cooperative membership (*n* = 301)		Cooperative membership (*n* = 143)		*β*_1__−_ *β*_2_	*t*-value
		Path coefficient (*β*)	*t*-value#	Path coefficient (*β*_1_)	*t*-value	Path coefficient (*β*_2_)	*t*-value		
Power imbalance	Relationship duration	0.03	0.60	0.01	0.02	0.02	0.27	0.01	0.10
	Sales volume	− 0.19	3.92***	− 0.04	0.65	0.11	1.10	0.15	1.34
Buyer/agent opportunism	Power imbalance	0.18	4.50***	0.17	3.23**	0.19	2.78**	0.03	0.31
Supplier financial performance	Power imbalance	− 0.03	0.64	− 0.15	3.21**	0.25	2.74**	0.40	4.37***
	Buyer/agent opportunism	− 0.08	1.64^a^	− 0.06	1.01	− 0.13	1.47	0.07	0.68
	Economic satisfaction	0.26	4.66***	0.37	5.71***	0.09	0.92	0.27	2.37*
	Social satisfaction	0.20	3.46***	0.10	1.36	0.32	3.58***	0.22	1.82
Economic satisfaction	Power imbalance	0.02	0.32	0.07	0.96	− 0.07	0.62	0.14	1.07
Social satisfaction	Power imbalance	− 0.11	2.33*	− 0.11	1.67^a^	− 0.09	0.89	0.02	0.18

^#^Based on 10,000 bootstrapping samples: ***significant at *p* < 0.001 level (two-tailed test); **significant at *p* < 0.01 (two-tailed test); *significant at *p* < 0.05 (two-tailed test)

^a^Significant at *p* < 0.05 (one-tailed test)

#### Financial Consequences of Power Imbalance and Opportunism

The objective behind the statement of the three hypotheses H4a, H4b and H4c is to explore the consequences of power imbalance and opportunism. H4a states that power imbalance is associated with reduced financial performance. We found support for H4a (*β* = − 0.24, *t* = 2.65, *p* < 0.01) (Table 5). H4b states that the effect of power imbalance on reducing financial performance is stronger for non-cooperative members than for cooperative members. To test H4b, we conducted a multigroup analysis based on data from study 2. The results of the multigroup analysis (Table 6) showed significant differences (β1 - β2 = 0.40, *p* < 0.001) between both groups, such that the effect of power imbalance on reducing financial performance is stronger for the non-cooperative members (*β* = − 0.15, *p* < 0.01) than for cooperative members (*β* = 0.25, *p* < 0.01). Concerning H4c, buyer/agent opportunism was found to be associated with reduced supplier financial performance (*β* = − 0.08, *t* = 1.64, *p* < 0.05) (Table 6).

#### Financial Consequences of Buyer Control of Price and Quality Standardization

**Fig. 5:**
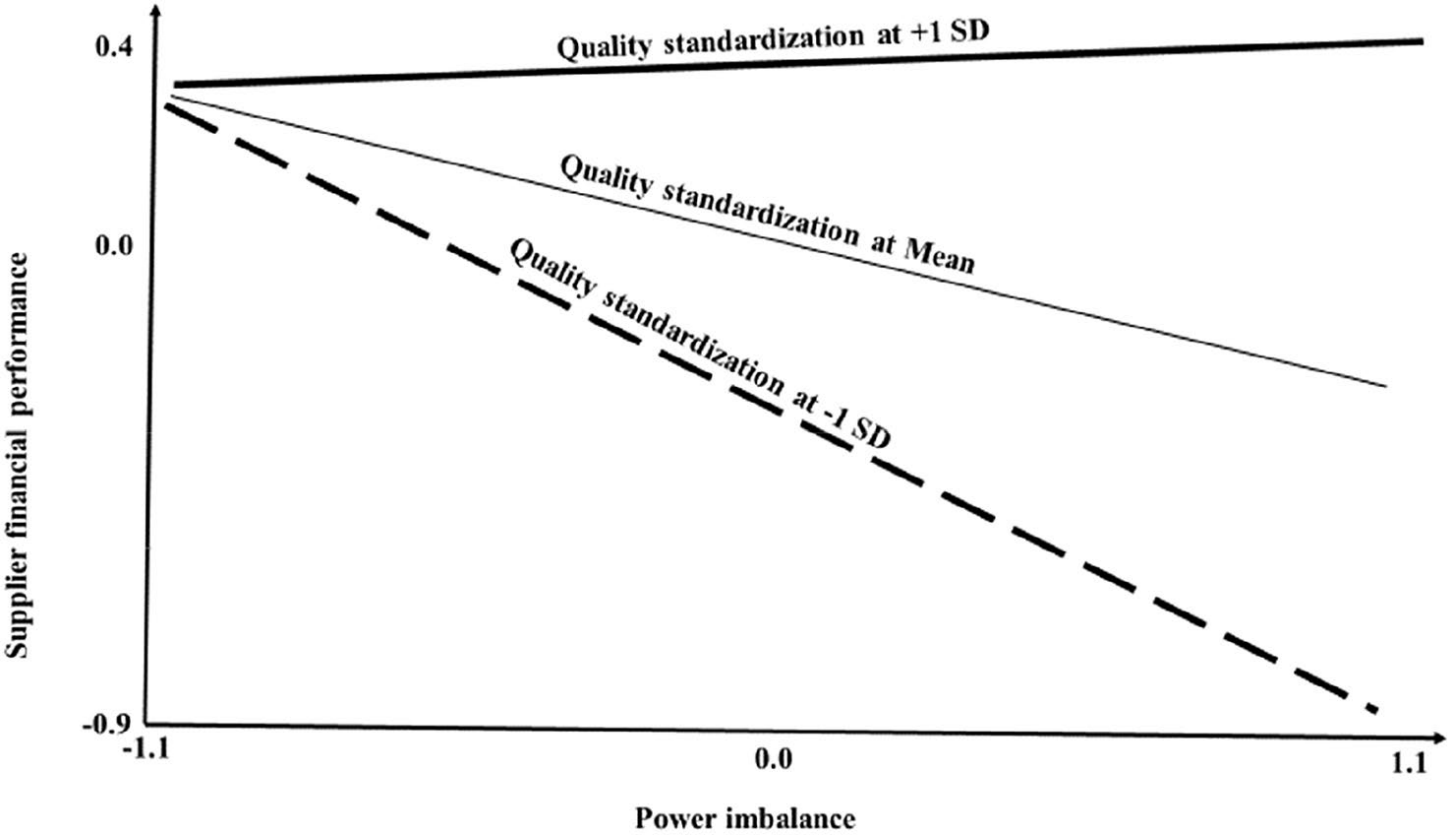
Simple slope analysis of the effect of power imbalance on supplier financial performance at various levels of quality standardization

From our analysis (Table 5), we found that power imbalance increased price control by the power source (*β* = 0.19, *t* = 2.24, *p* < 0.05). We suggest that buyer control of price is associated with reduced financial performance (H5a), while buyer control of quality in terms of quality standardization is associated with higher financial performance (H5b). We found support for both H5a (*β* = − 0.18, *t* = 2.69, *p* < 0.01) and H5b (*β* = 0.28, *t* = 2.76, *p* < 0.01). For H5c, we hypothesized that the effect of power imbalance on financial performance is moderated by quality standardization. The objective behind H5c was to determine the role played by decision control in the association between imbalance in power and reward. Our analysis shows that decision control regarding quality standardization can enhance the coordinative role of power in improving performance. Hence, we found support for H5c (*β* = 0.27, *t* = 3.17, *p* < 0.01) (see Table 5). We illustrate the test for H5c with a simple slope analysis, as shown in Fig. 5. At low levels of quality standardization, power imbalance reduces financial performance.

#### Linking Psychosocial Satisfaction and Collective Action with Reward

From our analysis (Table 5), we found that power imbalance decreased economic satisfaction (*β* = − 0.18, *t* = 1.92, *p* < 0.05). We propose that economic satisfaction is associated with increased financial performance (H6a) and suggest that the effect of economic satisfaction on financial performance is stronger for cooperative members than for non-cooperative members (H6b). We found support for the positive effect of economic satisfaction on financial performance (*β* = 0.43, *t* = 3.49, *p* < 0.001) (Table 5). The multigroup analysis, however, does not support H6b. Though we found significant differences (*β*_1_ − *β*_2_ = 0.27, *p* < 0.05) between the two groups, the effect of economic satisfaction on financial performance was found to be stronger for non-cooperative members (*n* = 301, *β* = 0.37, *p* < 0.001) than for cooperative members (*n* = 143, *β* = 0.09, p > 0.05) (Table 6).

## Discussion

Understanding how power asymmetry can provide a conducive environment for unethical behaviours is important in managing buyer–seller relationships in contemporary supply chains. Suppliers in captive agri-food supplier–buyer exchange relationships are highly dependent on buyers while having limited alternative outbound supply/marketing options. These suppliers are also exposed to performance risk and exploitation (Dadzie et al., 2018). We highlight the theoretical contributions and managerial implications of the study.

### Theoretical Contributions

Though a substantial number of studies have looked at the role of power and dependency in business exchanges and relationships, the role that power and dependency play in the dark side phenomenon is less understood. This implies that the mechanisms of the dark side phenomenon are not fully explained, and therefore our knowledge of these negative business practices is still insufficient. Our study presents several contributions to fill the knowledge gap. The study provides empirical evidence of the moderating role of transaction volume between supplier dependency and power imbalance consistent with the literature (Anderson & Weitz, 1989; Geyskens et al., 1996). Besides, our findings show that asymmetric power relations have a negative impact on the gains of the other party in the exchange and this is further exacerbated by dependency. This is particularly true when there are few alternatives for the dependent partner, such that the relationship becomes a captive one and prone to exploitation, consistent with the literature.

The effect of switching costs in such captive relationships is equally acknowledged in the literature, highly cited as the cause and most often assumed to be a linear relationship. Our contribution to the literature shows switching costs impact in imbalanced business relationships to be curvilinear. Similarly, the association between power imbalance and opportunism has been much cited (e.g. Grandinetti, 2017; Hawkins et al., 2008; Tangpong et al., 2015), but with little evidence on whether this association is linear or curvilinear. Our study reaffirms the linear association and shows that the effect of power imbalance on opportunism could also be curvilinear.

The study also provides empirical evidence to show the consequences of the dark side phenomenon. Power imbalance is associated with reduced financial performance of the weaker partner (Chicksand, 2015; Griffith et al., 2006; Hingley, 2005a). The concept of collective action refers to an action taken by a group of people with the aim to achieve a common goal and to improve their social or economic situation. Collection action is differentiated by a group/joint action and decision instead of that of an individual. Cooperative supply and marketing associations are typical examples. Our findings show significant differences between cooperative and non-cooperative groups in relation to the impact of power imbalance on financial performance. The theoretical implication is that the negative impact of the dark side phenomenon could be averted to a greater extent with group/joint action and concerted effort.

### Managerial Implications

Based on the research context, the cocoa agri-food supply market, the suppliers are heavily dependent on buyers and their agents. The impact of dependency on the power imbalance was found to be contingent on transaction volume, while the effect of power imbalance on financial performance was found to be moderated by supplier dependency. The implication is that small suppliers’ participation in collective action through supply and marketing cooperatives can help offset the asymmetrical power relations and improve individual and group welfare (Fischer & Qaim, 2012, 2014). Power can be used to control rewards to disadvantage the other party in the exchange; however, group action can provide a powerful voice for such disappointing incidents, relationship problems, and problematic business relationships. Collective action is a constructive group effort to change objectionable relationship conditions with the intent to improve conditions.

We draw on Dabholkar et al. (1994, p. 133), 'the interactions through which the terms of exchange are worked out are often referred to as the negotiation process'. Using the theory of negotiation (bargaining) behaviour (Clopton, 1984; Dabholkar et al., 1994; Perdue et al. 1986; Pruitt, 1981), negotiation behaviour can be classified along two dimensions of ''time'' and ''gain'' perspectives. Four categories of negotiation behaviour are identified: competitive, command, coordinative and cooperative. However, the terms ''cooperative'' and ''coordinative'' are used interchangeably in the literature. Competitive negotiation behaviour emphasizes individual short-term gain where parties maximize their outcomes. Command behaviour seeks out to maximize individual gain; however, the specific strategies are less 'coercive' than competitive bargaining behaviour.

Coordinative strategies focus on the long-term joint gain, while the cooperative approach is characterized by the short-term joint gain (Dabholkar et al., 1994). Though the above-negotiating strategies apply mostly to dyadic exchanges, it can also apply to network relations although more complex. Exchange relations between cocoa suppliers and lead firms can be classified along the two dimensions of short/long-term and individual/joint gain. Individual cocoa farmers dealing with lead firms and the government parastatal organization (Ghana Cocoa Board) have less bargaining power than if organized as cooperatives.

An important strategy for protecting firms from unethical behaviour is information sharing (Eckerd & Hill, 2012). The sharing of records and accounts to reduce the information asymmetry between buyer and supplier firm can help stimulate trust. This action is a critical obligation as emerging markets liberalize their agri-food supply markets and exporters compete for market share for cocoa and coffee in the originating countries (Dadzie et al., 2018). Others have recommended accredited ethical purchasing agents (Buxton & Vorley, 2012), where lead firms dealing with small suppliers can educate, train and align the objectives, incentives and motivations of their sourcing managers, frontline staff and intermediaries to be ethical with suppliers (Vorley & Thorpe, 2014). Building ethical and mutually beneficial relationships between small dependent suppliers and lead firms is an important strategy to reduce the negative consequences of exploitative business relationships and to control opportunism (Glavee-Geo et al., 2020).

Corporate ethical values (CEV) and formal ethical infrastructure are key features of an organization that can be relied on to ensure employees' attitudinal and behavioural change. This calls for clear ethically responsible purchasing practices, codes of conduct, and enforceable company policies (Saini, 2010). However, ethical codes are not enough. Hyman et al. (1990) suggests the need for a checklist for evaluating managerial decisions, and to improve the chances of being ethical. CEVs represent the amount of attention afforded to ethical issues by the firm, and the degree to which the firm behaves ethically (Hunt et al., 1989). A firm's formal ethical infrastructure (FEI) is characterized by formal communication, recurrent communication, formal surveillance, and formal sanctions and has been found to influence employee’s moral awareness (Hawkins et al., 2013; Rottig et al., 2011). The ethical infrastructure of an organization includes a range of organizational systems that guide ethical decision-making (Hawkins et al., 2013). Hence the communication of ethical standards to employees engenders moral behaviour (Eckerd and Hill, 2012).

Laudable as some of the strategies may be, more so when most of the initiatives are at the lead firm's benevolence, the challenge is how can resistant lead firms be convinced? Global supply chains have become overly complex and more vulnerable to disruptions with large unanticipated consequences (Fahimnia et al., 2015). Also, notwithstanding major disruptions either human-made or natural (e.g. COVID-19) and supply risk caused by several sources of inherent uncertainties such as demand fluctuations, supply capacity changes, lead time variability, and exchange rate volatility (Fahimnia et al., 2015), firms face enormous pressure and responsibility to deliver consistently. The need to ensure consistent supply and be resilient implies that firms have no choice than to be ‘compliant’, socially responsible and enforce ethical behaviour among employees (sourcing and purchasing agents, intermediaries, and staff).

Supplier scarcity has been cited (Schiele et al., 2012) as an important reason for supplier resource mobilization and supplier development. Firms compete not only on the sales market but also on the supply market since ‘really good’ suppliers are scarce (Cordón & Vollmann, 2008; Schiele et al., 2012, p. 1178). Suppliers can actively differentiate among competing buyers based on their ethical standards such that ethical behaviour can be a differentiating attribute and cue. Thus, corporate ethical values can be used as a ‘discriminating’ signal to communicate a firm’s attractiveness to suppliers and beat competing buyers (especially regarding strategic products/services). ‘Since more and more sales revenue comes from purchased items, it becomes more critical to get the best—ahead of your competitors’ (Cordón & Vollmann, 2008, p. 14). Being ethical is being ‘smart’. The behaviours and activities (regarding ethicality) of the buying company become a key means of influencing suppliers' resource mobilization. It is also a means of communicating the firm's corporate reputation to other stakeholders (government agencies, customers, civil society organizations, and the wider public) and reinforcing the firm's social responsibility. Besides, unethical opportunistic behaviour can also be a costly problem within the organization and lead to substantial inefficiencies and reputational challenges.

The dark side phenomenon leads to challenges that inhibit creativity, innovation, and value creation (Abosag et al., 2016, p. 5). Value formation is not only associated with value co-creation but also with value co-destruction (Echeverri & Skålén, 2011). The co-destruction of value is one of the downsides of value co-creation, and this has ethical implications for business relations. Besides, power imbalance impedes supply chain innovation and improvement by the less dominant partner. Such a power imbalance can lead to the use of coercive power by the more dominant buyer. Small suppliers must devote more resources to ensuring the continuation of business relationships with powerful buyers at the expense of supply chain innovations (Matanda et al., 2016). Our study suggests that buyer control of price is associated with reduced financial performance, while buyer control of quality in terms of quality standardization is associated with higher financial performance. In addition, we found that decision control regarding quality standardization can enhance the coordinative role of power in improving performance. The implication is that the use of power for coordination can be more effective if this is done within an environment characterized by mutuality, honesty, empathy, and fairness (Woiceshyn, 2011).

Finally, the positive impact of economic satisfaction on financial performance was found to be stronger for non-cooperative members than for cooperative members. This could be explained by the fact that cooperative members expect more gains from their exchanges than non-cooperative members. Cooperative members invest more time and effort in collective action. The implication is that although collective action may not solve all challenges emanating from problematic business relationships, joint/collective action is one of the most viable options in opposition to the negative impact of the dark side. For example, fair trade initiatives that strive for more equitable sharing of profits among the members are typical examples of sustainable collaborative supply chains (Drake and Schlachter, 2008).

## Conclusion, Limitations, and Further Research

Our research highlights the following:

- How dependency and high switching costs in captive supplier–buyer exchanges influence perceptions of power imbalance.
- The conditions under which power imbalance provides ideal situations for unethical behaviours.
- The consequences of power imbalance, opportunistic behaviour, and decision control.
- The theoretical contributions and managerial implications.

Dependency and high switching costs in captive supplier–buyer exchanges influence perceptions of power imbalance. The impact of dependency on power imbalance is contingent on transaction volume, such that as the volume of transactions between trading partners increases, dependency leads to power imbalance. This eventually leads to opportunities for exploitation of the weaker partner.

Our study has provided empirical evidence of the unique relationships between the main constructs under study (see Table 7) and discussed the theoretical contributions of the study, briefly summarized into five main propositions as follows. (1) Asymmetric power relations’ negative impact on the gains of the other party in the exchange is exacerbated by dependency. (2) The consequences of power imbalance are opportunism and low financial reward. (3) The effect of power imbalance on opportunism is not only linear but could also be curvilinear. (4) The negative impact of power imbalance in reducing financial performance is exacerbated without joint effort and collective action. (5) Not only does decision control in asymmetric relations lead to the unfair distribution of rewards, but it also provides the conditions for exploitation and unethical behaviour. We have also provided managerial insights in relation to control, monitoring, collective action, fairness, transparency, ethical sourcing/purchasing and chain governance. We emphasize the need for businesses to invest in strategies and procedures that protect suppliers against detrimental actions and behaviours by boundary spanning personnel and intermediaries.

**Table 7:** Summary of findings

Associations	Uniqueness of hypothesized association	Sign	Results
Key antecedents of power imbalance (shadow of dark side)			
Supplier dependency → Power imbalance (H1a)	Moderated by transaction volume	**+**	Strongly supported
Switching costs → Power imbalance (H2a)	Direct effect and linear	**+**	Not supported
Switching costs → Power imbalance (H2b)	Curvilinear (U shape)	**+**	Supported
Consequences of power imbalance (negative—dark side)			
Power imbalance → Financial performance (H1b)	Moderated by supplier dependency	**−**	Supported
Power imbalance → Buyer/agent opportunism (H3a)	Direct effect and linear	**+**	Supported
Power imbalance → Buyer/agent opportunism (H3b)	Curvilinear (inverted U shape)	**−**	Strongly supported
Power imbalance → Financial performance (H4a)	Direct effect and linear	**−**	Supported
Power imbalance → Financial performance (H4b)	Moderated by cooperative/collective action	**−**	Ncoop>Coop**—**strongly supported
Consequences of opportunism (negative—dark side)			
Buyer/agent opportunism → Financial performance (H4c)	Direct effect and linear	**−**	Supported
Negative consequences of decision control (negative—dark side)			
Buyer control of price → Financial performance (H5a)	Direct effect and linear	**−**	Supported
Positive consequences of decision control (positive—dark side)			
Quality standardization → Financial performance (H5b)	Direct effect and linear	**+**	Strongly supported
Power imbalance → Financial performance (H5c)	Moderated by quality standardization	**+**	Strongly supported
Positive consequences of economic satisfaction (positive—light side)			
Economic satisfaction → Financial performance (H6a)	Direct effect and linear	**+**	Strongly supported
Economic satisfaction → Financial performance (H6b)	Moderated by cooperative/collective action	**+**	Coop>Ncoop**—**not supported

*Ncoop* non-cooperative membership of supplier, *Coop* cooperative membership of supplier

We acknowledge some limitations of this study, which can be addressed in future research. Firstly, we considered the suppliers’ perspective, which is just one side of the buyer–supplier dyad; however, perceptions may vary across the dyad (John & Reve, 1982), such that suppliers and buyers may have different perceptions and interpretations of what goes on in the dyad. Thus, future research could focus on the interdependent and simultaneously interacting perspectives of suppliers and buyers. Secondly, this study investigated power asymmetry using the cocoa industry in a developing economy as the setting. Hence, the findings may not apply to all industries. However, the results can be extended to industries with similar power relations such as the agribusiness (coffee, tea, horticulture—fruits, vegetables, floral), textiles and garment industries, and the grocery/retail sectors. Some of the cooperative associations studied in this article may vary regarding buyer–supplier relations in industrial settings in the developed economies due to institutional differences. Further replication studies involving small to medium-sized supply companies in asymmetrical industrial supplier–buyer relationships in developed economies will help provide more support and confirmation of the various hypothesized associations in the present study.

Thirdly, the research context and setting's findings assume homogenous outcomes, benefits, and impact for all cooperative members. Our study did not take into consideration the number of cooperative associations. Hence, we were not able to explore if the findings relating to the negative impact of power imbalance on financial performance and the positive effect of economic satisfaction on financial performance differs across the many cooperatives. Future studies should consider how the impact of power imbalance on financial performance as well as the positive effect of economic satisfaction on financial performance differs across the many cooperatives and its corresponding ethical considerations.

Besides, we did not explore the motivations and expectations about cooperative membership or why some farm enterprises did not join the cooperatives. This provides more options for future research about the motivations and expectations of cooperative membership and its impact on value creation and co-destruction. In addition, power, dependence, and exploitation can occur in cooperative relationships and joint actions even among suppliers. Conflict and power dependence can occur in cooperatives.

Our study focused on joint action as one of the possible solutions to address exploitation. Collective action is one of the strategies to tackle this ethical problem. However, other viable options have not been considered in the present study, presenting future research opportunities. Finally, the paper addressed the supplier–buyer perspective and not the buyer–supplier or supplier–supplier exchanges and perspectives. Thus, there is a need for more studies from other perspectives apart from the suppliers. Future studies on power, dependence and exploitation in joint/collaborative actions can present new insights on the dark side phenomenon.
